# Oncolytic therapy with vaccinia virus carrying IL-24 for hepatocellular carcinoma

**DOI:** 10.1186/s12985-022-01779-1

**Published:** 2022-03-15

**Authors:** Lili Deng, Xue Yang, Yuedi Ding, Jun Fan, Ying Peng, Dong Xu, Biao Huang, Zhigang Hu

**Affiliations:** 1 NHC Key Laboratory of Nuclear Medicine, Jiangsu Key Laboratory of Molecular Nuclear Medicine, Jiangsu Institute of Nuclear Medicine, Wuxi, 214063 China; 2 Wuxi Children’s Hospital, Wuxi People’s Hospital Affiliated to Nanjing Medical University, Wuxi, 214023 China; 3 School of Life Science, Zhejiang Sci-Tech University, Hangzhou, 310018 Zhejiang China

**Keywords:** Hepatocellular carcinoma, Oncolytic vaccinia virus, Gene therapy, Interleukin-24, Apoptosis

## Abstract

**Background:**

Hepatocellular carcinoma (HCC) is a highly refractory cancer associated with increasing mortality, which currently lacks effective treatment options. Interleukin-24 (IL-24) is a novel tumor suppressor cytokine that can selectively induce cancer cell apoptosis, and it has been utilized as a cancer gene therapy strategy. The vaccinia virus is a promising strategy for cancer therapy, owing to its direct viral lytic effects, as well as a vehicle to overexpress therapeutic transgenes.

**Methods:**

We constructed a recombinant oncolytic vaccinia viruse (VG9-IL-24) based on vaccinia virus Guang9 (VG9) harboring the *IL-24* gene. In vitro*,* we assessed the replication of VG9-IL-24 in HCC cell lines and normal liver cells and evaluated the cytotoxicity in different cell lines; then, we determined the expression of IL-24 by RT-PCR and ELISA. We examined apoptosis and cell cycle progression in SMMC-7721 cells treated with VG9-IL-24 by flow cytometry. In vivo, we established the SMMC-7721 xenograft mouse model to evaluate the antitumor effects of VG9-IL-24.

**Results:**

In vitro*,* VG9-IL-24 efficiently infected HCC cell lines, but not normal liver cells, and resulted in a high level of IL-24 expression and significant cytotoxicity. Moreover, VG9-IL-24 induced an increase in the proportion of apoptotic cells and blocked the SMMC-7721 cell cycle in the G2/M phase. In vivo*,* tumor growth was significantly suppressed and the survival was prolonged in VG9-IL-24-treated mice.

**Conclusions:**

Vaccinia virus VG9-mediated gene therapy might be an innovative treatment for cancer with tumor-specific lysis and apoptosis-inducing effects. VG9-IL-24 exhibited enhanced antitumor effects and is a promising candidate for HCC therapy.

## Background

Hepatocellular carcinoma (HCC) is an aggressive primary liver cancer which is resistant to current chemotherapy and radiotherapy. Despite the clinical approval of sorafenib (a multi-kinase inhibitor drug) for the treatment of advanced HCC, minimal improvements have been made to overall therapeutic outcomes, and the survival benefit has been investigated, as it is also associated with significant toxicity and drug resistance [1, 2].

Oncolytic vaccinia viruses have emerged as a novel therapeutic treatment for cancer owing to the inherent capacity to infect and replicate within tumor cells, resulting in virus progeny production, tumor cells lysis, and spread to adjacent and distant tumor cells [3]. Tumor destruction is mediated not only by cell lysis but can also be enhanced by engineering of therapeutic genes that modulate the tumor microenvironment or tumor cells directly. With the replication of vaccinia virus, the copies of genes harbored by virus are also increased, leading to higher expression levels in tumor tissues. JX-594, a Wyeth strain modified by granulocyte microphage colony-stimulating (GM-CSF), was found to eradicate lung metastases from liver tumors in rabbits [4] and had antitumor effects both at the injection site and in distant non-injected tumors in advanced HCC patients [5]. The Lister strain GLV-1h168 has also showed antitumor efficacy in HCC [6], and such efficacy was not affected in sorafenib-resistant HCC cell lines, which would therefore be beneficial for patients for whom treatment with sorafenib have failed [7].

In this study, we constructed a recombinant oncolytic vaccinia virus based on vaccinia strain Guang9 (VG9), which was derived from Chinese Tian Tan strain of vaccinia virus (VTT) and has been demonstrated to be more attenuated [8]. The therapeutic gene engineered here was interleukin-24 (IL-24), which is a novel tumor suppressor cytokine that induces apoptosis in various tumor cells but has no significant cytotoxicity to normal cells [9]. The traditional delivery of IL-24 by liposomes or replication-defective adenovirus [10–12] cannot target tumor cells, which limits its value on cancer gene therapy. Here, the oncolytic VG9 strain carrying the *IL-24* gene (VG9-IL-24) was constructed, and the antitumor effects and therapeutic potential on HCC were evaluated both in vitro and in vivo.

## Methods

### Cell lines

HCC cell lines including SMMC-7721, HepG2, and Hep3B and the normal human liver cell line HL-7702 were purchased from Cell Bank of Shanghai Institutes for Biological Sciences of the Chinese Academy of Sciences (Shanghai, China). African green monkey kidney epithelial cell lines BSC-40 and Vero cells were purchased from the American Type Culture Collection (ATCC; Rockville, MD, USA). All cell lines were cultured in DMEM or RPMI-1640 (Thermo Fisher Scientific, Inc., Waltham, MA, USA) supplemented with 10% fetal bovine serum (Gibco-BRL, Grand Island, NY, USA), 2 mmol/L glutamine, 50 U/mL penicillin, and 50 μg/mL streptomycin. All cells were maintained at 37 °C in a humidified incubator with 5% CO_2_.

### Recombinant vaccinia viruses

**Fig. 1:**
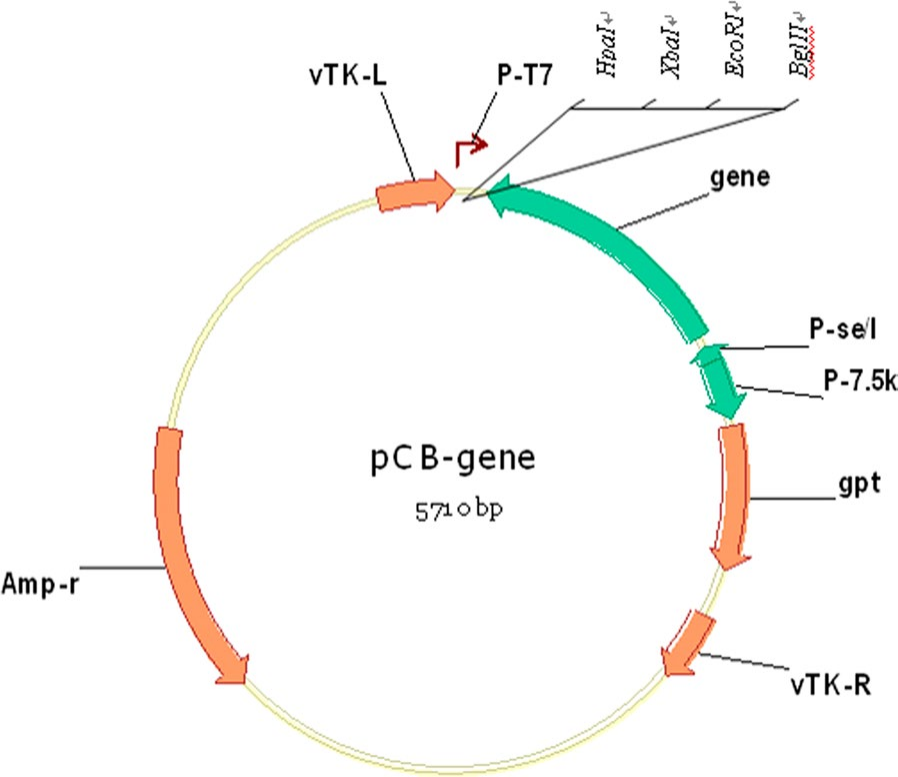
Schematic illustration of plasmid pCB. vTK-L, vTK-R: portions of the vaccinia thymidine kinase (TK) gene; P-se/I, P-7.5K: vaccinia synthetic early promoter; P-T7: T7 RNA polymerase promoter; gpt: xanthine-guanine phosphoribosyltransferase gene

The vaccinia VG9 strain was obtained from National Institutes for Food and Drug Control (NIFDC, Beijing 100050, China). The recombinant vaccinia virus was constructed via homologous recombination between VG9 and the shuttle plasmid pCB (gifted from Professor Liu, Institute of Biochemistry and Cell Biology, Shanghai Institutes for Biological Sciences, The Graduate School, Chinese Academy of Sciences). The pCB plasmid was flanked by portions of the vaccinia thymidine kinase (TK) gene (vTK-L, vTK-R), which facilitates homologous recombination into this locus (Fig. 1). The human IL-24 gene (Gene ID: 11009; full-length cDNA was purchased from Sino Biological Inc., Beijing, China) or enhanced green fluorescent protein (EGFP) gene was inserted into TK locus and was under the control of the vaccinia synthetic early/late promoter. Recombinants were selected in Vero cells via xanthine-guanine phosphoribosyltransferase (XGPRT) selection [13]. VG9-EGFP was constructed as a control and conserved in our laboratory [14]. All recombinant vaccinia viruses were purified in sucrose gradient and virus stocks were titrated on BSC-40 cells to confirm pfu/mL prior to use.

### Fluorescence microscopy

SMMC-7721 and HL-7702 cells were infected with 0.1 multiplicity of infection (MOI) of VG9-EGFP and observed under a fluorescence microscope after infection for 48 h.

### Viral replication assay

The replication ability of VG9-IL-24 was investigated in HCC cell lines and HL-7702 cells at 0.1 MOI. After incubation in growth medium containing 2% fetal bovine serum for 2 h, cells were then incubated in complete growth medium. Cells and supernatant were harvested at 48 h and lysed during three cycles of freezing and thawing. Viral titers were determined on BSC-40 cells.

### MTT assay

The HCC cell lines and HL-7702 cells seeded in 96-well plates were infected with different concentrations (0.01, 0.1, 1, and 10 MOI) of VG9-IL-24 for 72 h. Cells without virus infection were used as controls. After incubation with MTT (Sigma, St. Louis, MO, USA) for 4 h, the medium was removed and the reaction was stopped with dimethyl sulfoxide (DMSO). The optical absorbance was measured at a wavelength of 490 nm by SpetraMax M5 microplate reader (Molecular Devices, Sunnyvale, CA, USA).

### IL-24 expression

SMMC-7721 cells grown in 6-well plates were infected with 0.1 MOI of VG9-IL-24. Cells were harvested 48 h after infection, and total RNA was extracted using TRIzol reagent (Invitrogen Life Technologies, Carlsbad, CA, USA). cDNA was obtained from the total RNA using the One Step RT-PCR kit (Promega, Madison, WI, USA) according to the manufacturer’s instructions. Primers used for the quantification of IL-24 and GAPDH mRNA were as follows: sense 5′-GCAACCCAGTCAAGAAAATGAG-3′, antisense 5′- AAGAATGTCCACTTCCCCAAG-3′ for IL24; sense 5′-AATCCCATCACCATCTTCCAG-3′, antisense 5′-AAATGAGCCCCAGCCTTC-3′ for GAPDH. PCR amplification and detection were carried out on an ABI Prism 7500 Sequence Detecction System (Applied Biosystems) with the following conditions: denaturation at 94 °C for 5 min; 30 cycles of denaturation at 94 °C for 30 s, annealing at 55 °C for 30 s, and extension at 72 °C for 30 s; and extension at 72 °C for 10 min to ensure full extension of the product. Relative mRNA levels were calculated using the ΔCt method [15]. All mRNA levels are normalized to GAPDH mRNA levels.

Supernatants and lysates were collected after VG9-IL-24 infection for 48 h and IL-24 levels were quantitatively determined with an enzyme-linked immunosorbent assay (ELISA) kit (R&D Systems Inc., Minneapolis, MN, USA) according to the manufacturer’s manual.

### Cell apoptosis detection

Morphological characteristics of apoptotic cells were observed based on Hoechst 33258 staining. HCC cell lines and HL-7702 cells were infected with VG9-IL-24 or VG9-EGFP for 24 h and PBS-treated cells were used as a negative control. Then, cells were incubated with Hoechst 33258 (Beyotime Biotechnology, China) for 30 min. The apoptotic morphological changes of cells were immediately observed under the Olympus IX51 fluorescence microscope.

The ratios of apoptotic cells were further determined by flow cytometric analysis using an Annexin V/propidium iodide (PI) apoptosis detection kit (Roche Applied Science, Germany). SMMC-7721 cells were harvested after treatment with VG9-IL-24, VG9-EGFP, or PBS for 12 h. Aliquots of cells were resuspended in 1 mL binding buffer mixed with 20 μL of fluorescein isothiocyanate (FITC)-labeled annexin V (0.5 μg/mL; as an early apoptotic marker) and 20 μL of PI (0.5 μg/ml; as a late apoptotic marker) according to the manufacturer’s instructions. After 10 min incubation in the dark at room temperature, flow cytometry (BD, FACSCalibur, USA) was performed immediately.

### Cell cycle analysis

SMMC-7721 cells seeded in 6-well plates were infected with VG9-IL-24, VG9-EGFP, or PBS for 48 h, and were then harvested and fixed in 70% cold ethanol overnight at − 20 °C. Cells were washed with PBS and resuspended in 50 μg/mL of PI solution. After incubation for 30 min in the dark at 37 °C, the treated cells were analyzed by flow cytometry. The percentages of G0/G1, S, and G2/M stage cells were quantified using Flow Jo Software (Tristar, CA, USA).

### Animal experiments

The animal experiments were performed according to the Declaration of Helsinki and were approved by the Institutional Animal Care and Use Committees (IACUC) of Jiangsu Institute of Nuclear Medicine (JSINM2010007). Female nude BALB/c mice (5–6 weeks old) were purchased from Shanghai Laboratory Animals Center (SLAC; Shanghai, China). They were housed under specific pathogen free conditions with a controlled temperature and humidity and a 12–12 h day-night light cycle. Mice were given free access to food and water.

A total of 18 mice were used in the study. Each nude mouse was subcutaneously injected with 2 × 10^6^ SMMC-7721 cells into the left armpit. When tumors reached 3–5 mm in diameter, mice were randomized into three groups using random the number table method (n = 6 in each group). A single injection of PBS (control group), 10^7^ PFU of VG9-IL-24 (VG9-IL-24 group) and 10^7^ PFU of VG9-EGFP (VG9-EGFP group) was administered intratumorally. Tumor growth was followed, and the tumor volume was calculated as [(width)^2^ × length] × 0.52. When tumors reached their maximal permitted size according to the animal regulations, mice were euthanized in a CO_2_ chamber at a concentration less than 40% with a flow rate of 50% chamber vol/min. Kaplan–Meier survival curves were plotted.

### Statistical analysis

Statistical analysis was performed by SPSS 19.0 software (SPSS Statistics, Inc., Chicago, IL, USA). Data were presented as the mean ± standard deviation (SD). One-way ANOVA analysis was employed to compare multiple groups. Survival analysis was performed using the method of Kaplan–Meier, and differences between curves were assessed using the log-rank test. *P* < 0.05 was considered statistically significant.

## Results

### VG9-IL-24 expression in HCC cell lines

**Fig. 2:**
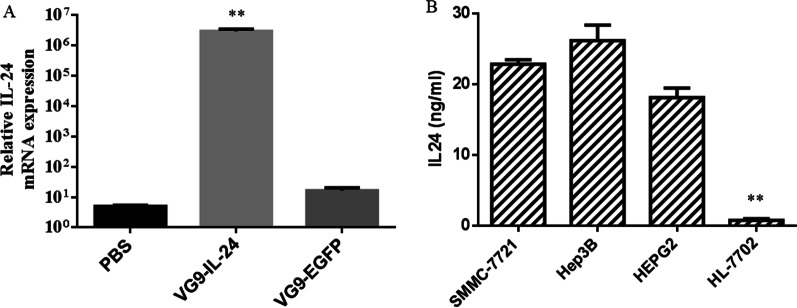
The expression of VG9-IL-24 mediated IL-24 in vitro. **A** RT-PCR analyzed transcription of exogenous IL-24 genes in SMMC-7721 cells after treatment with PBS, VG9-IL-24, and VG9-EGFP. **B** The expression of exogenous IL-24 protein was analyzed by ELISA. Each bar represents the mean ± SD (n = 3). **P < 0.01

To test whether VG9-IL-24 can mediate the transcription of the exogenous IL-24 gene, SMMC-7721 cells were treated with PBS, VG9-IL-24, and VG9-EGFP respectively. After treatment for 48 h, the mRNA level of IL-24 was significantly detected only in cells infected with VG9-IL-24 (Fig. 2A). To further evaluate the expression of exogenous IL-24, protein in supernatants and lysates from HCC cell lines and normal liver cells infected with VG9-IL-24 were harvested and quantified. ELISA results showed that the concentrations of IL-24 protein were higher in HCC cells, while those were lower in normal HL-7702 cells (Fig. 2B). No IL-24 production was detected in cells treated with PBS or VG9-EGFP (data not shown).

### Oncolytic activity of VG9-IL-24 in vitro

**Fig. 3:**
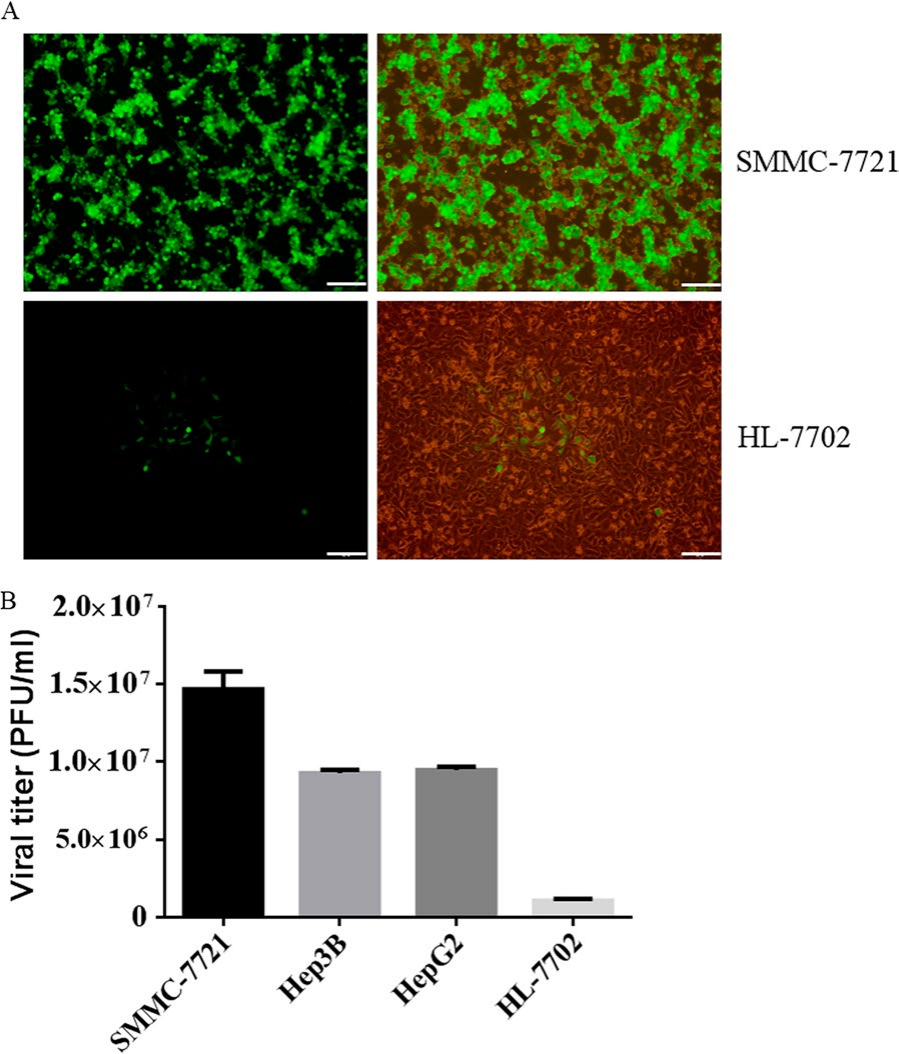
Oncolytic activity of VG9-IL-24 in vitro. **A** The proliferation of VG9-EGFP in SMMC-7721 cells and HL-7702 cells. The viral replication was monitored under the fluorescence microscope after infection for 48 h. Bar: 20 μm. **B** VG9-IL-24 selectively replicated in tumor cells. HCC cells and HL-7702 cells in 12-well plates were infected with 0.1 MOI of VG9-IL-24 and samples were collected at 48 h. Each bar represents the mean ± SD (n = 3)

To determine the ability of recombinant vaccinia virus to selectively replicate in tumor cells, we first tested the growth of VG9-EGFP in malignant and normal cells. As shown in Fig. 3A, the green fluorescence in SMMC-7721 cells was pervasive and integrated presence and the EGFP expression was stronger, indicating that the virus replicated efficiently in HCC cells. In contract, EGFP expression in normal cells (HL-7702) was lower and the green fluorescence was scattered presence, indicating no significant viral replication.

Then, we evaluated the replication ability of VG9-IL-24 in various HCC cell lines and normal liver cells. As shown in Fig. 3B, VG9-IL-24 was efficiently replicated in various HCC cell lines, and SMMC-7721 cells exhibited a peak titer > 1200-fold higher at 48 h post-infection. In contrast, replication of VG9-IL-24 in normal cells (HL-7702) was not significant; the viral titer was only 28-fold higher after infection for 48 h.

**Fig. 4:**
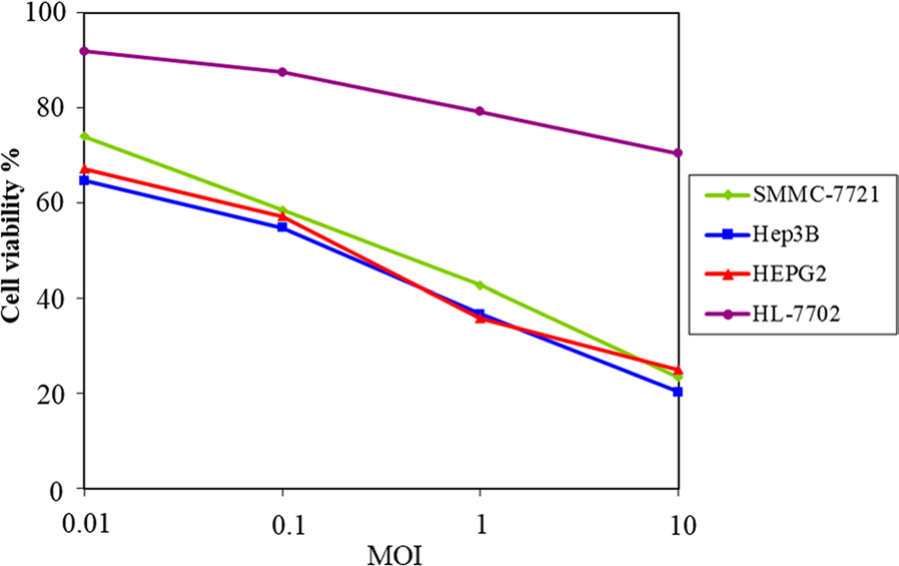
VG9-IL-24 selectively induced cytotoxicity in tumor cells. HCC cell lines and normal liver cell line were infected with VG9-IL-24 at various MOIs and cell viability was measured by MTT assay after infection for 72 h

To further assess the kinetics of cytotoxicity induced by VG9-IL-24, malignant and normal cells were infected with VG9-IL-24 at different dosages and cell viability was determined by MTT assays. As shown in Fig. 4, HCC cell lines showed significant sensitivity to VG9-IL-24, which killed all cancer cell lines in a dose-dependent manner, whereas VG9-IL-24 had little cytotoxic effects on normal cells.

Together, these data suggest that VG9-IL-24 is a tumor-selective oncolytic virus and has strong tumoricidal activity in HCC cells.

### VG9-IL-24-mediated apoptosis in HCC cells

**Fig. 5:**
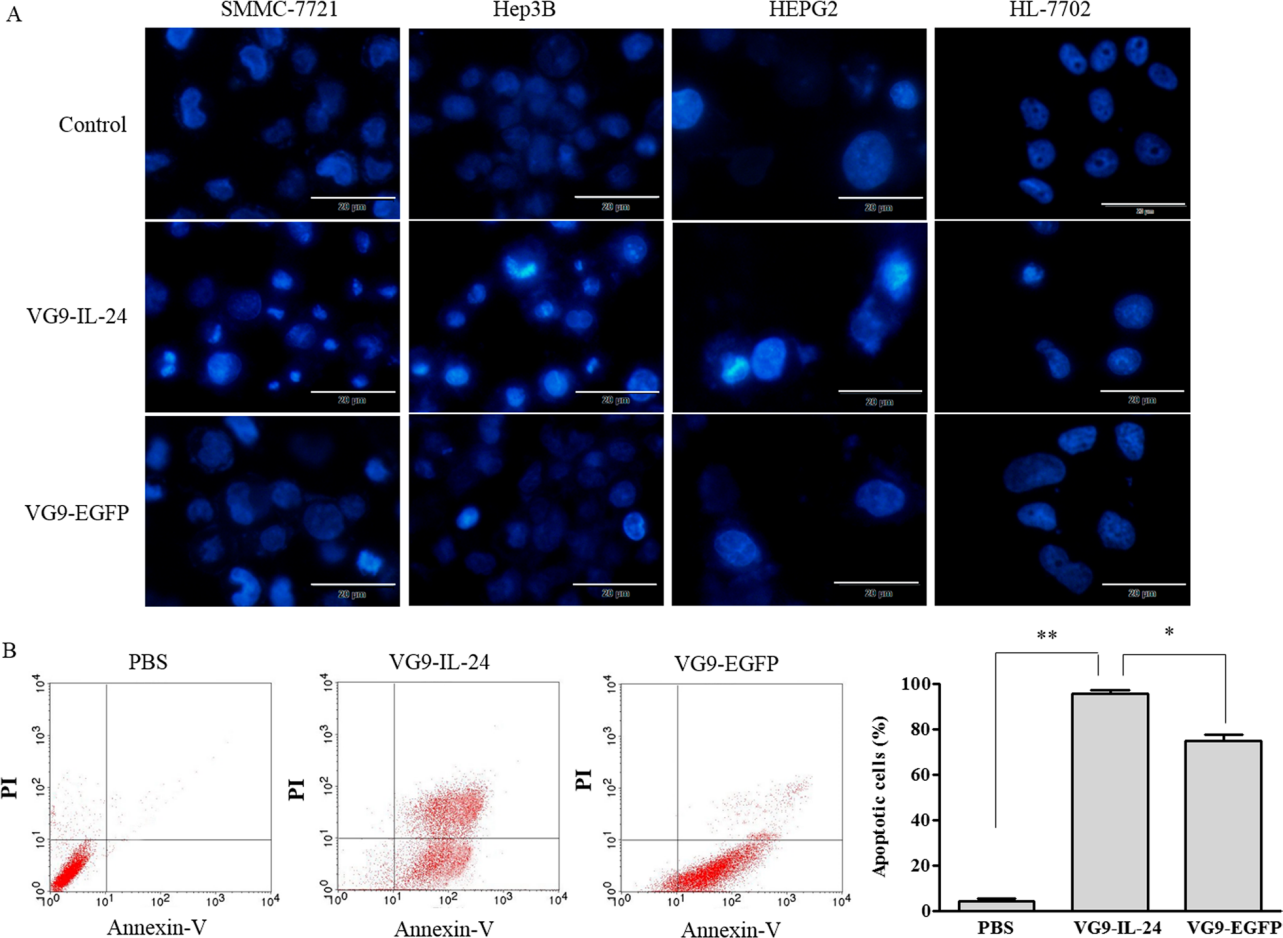
VG9-IL-24 induced apoptosis of hepatocellular carcinoma cells. **A** SMMC-7721, Hep3B, HepG2 and HL-7702 cells treated with PBS, VG9-IL-24 or VG9-EGFP for 24 h were stained with Hoechst 33258 to visualize morphological changes. Chromatin condensation, nuclear shrinkage, or fragmentation were observed in virus-treated groups but not in normal cells. Bar: 20 μm. **B** The percentage of apoptotic cells was determined by flow cytometry. SMMC-7721 cells treated with PBS, VG9-IL-24 or VG9-EGFP were harvested after 12 h and stained with FITC-labeled annexin V and PI and immediately analyzed by flow cytometry. Each bar represents the mean ± SD (n = 3). *P < 0.05 versus VG9-EGFP group; **P < 0.01 versus PBS (Control) group

To confirm whether VG9-IL-24 induced apoptosis in HCC cells, SMMC-7721, HepG2, Hep3B and the normal cell line HL-7702 were analyzed for morphological alterations indicative of apoptosis by Hoechst 33258 staining. Results showed that VG9-IL-24 induced apoptosis in various HCC cell lines with the observation of nuclear fragmentation and chromatin clumping, but no evident apoptotic changes occurred in normal HL-7702 cells (Fig. 5A). VG9-IL-24-mediated apoptosis in HCC cells was increased relative to that in the VG9-EGFP group.

Annexin-V and PI staining assays coupled with flow cytometry further quantified the effect of the various treatments on apoptosis in SMMC-7721 cells (Fig. 5B). The proportion of apoptotic cells increased significantly in VG9-IL-24-infected cells compared to that with other treatments (*P* < 0.01 compared with PBS group; *P* < 0.05 compared with VG9-EGFP group).

### Cell cycle analysis

**Fig. 6:**
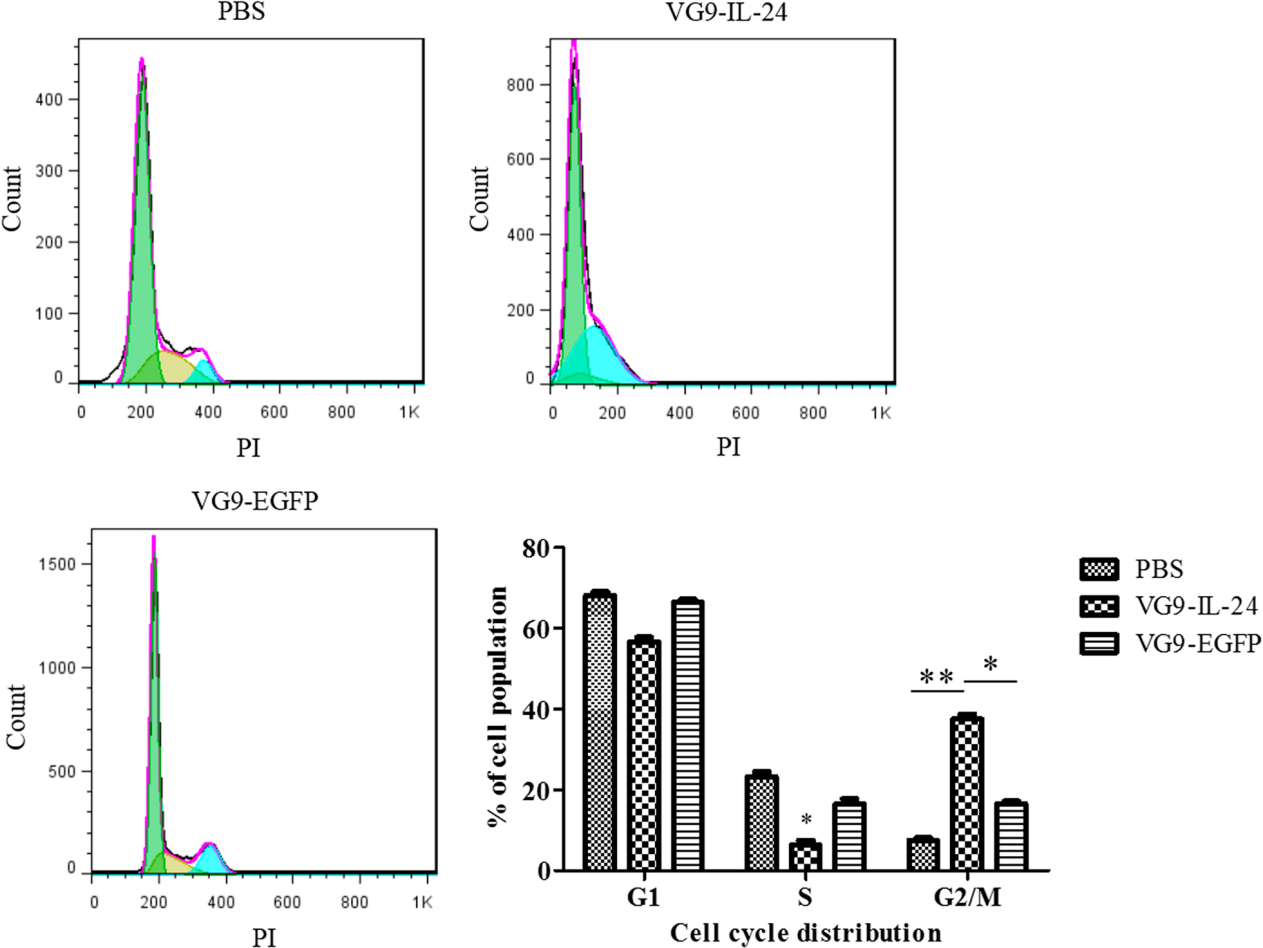
Cell-cycle analysis by flow cytometry. SMMC-7721 cells treated with PBS, VG9-IL-24 or VG9-EGFP were harvested after 48 h and stained with PI. Cell cycle distribution was analyzed by flow cytometry and the percentage of cell-cycle phases was analyzed by Flow Jo Software. Each bar represents the mean ± SD of three independent experiments. *P < 0.05; **P < 0.01

To determine whether VG9-IL-24-mediated cell death was related to cell cycle arrest, cell cycle analysis with single PI staining and flow cytometry was performed. Results showed that VG9-IL-24 induced G2/M cell-cycle arrest in SMMC-7721 cells compared to that with PBS and VG9-EGFP (Fig. 6; *P* < 0.05). The proportions of cells in the G2/M phase were (7.42 ± 0.70)%, (37.50 ± 1.26)% and (16.53 ± 0.61)% in PBS, VG9-IL-24 and VG9-EGFP groups, respectively.

### Antitumor efficacy of VG9-IL-24 in vivo

**Fig. 7:**
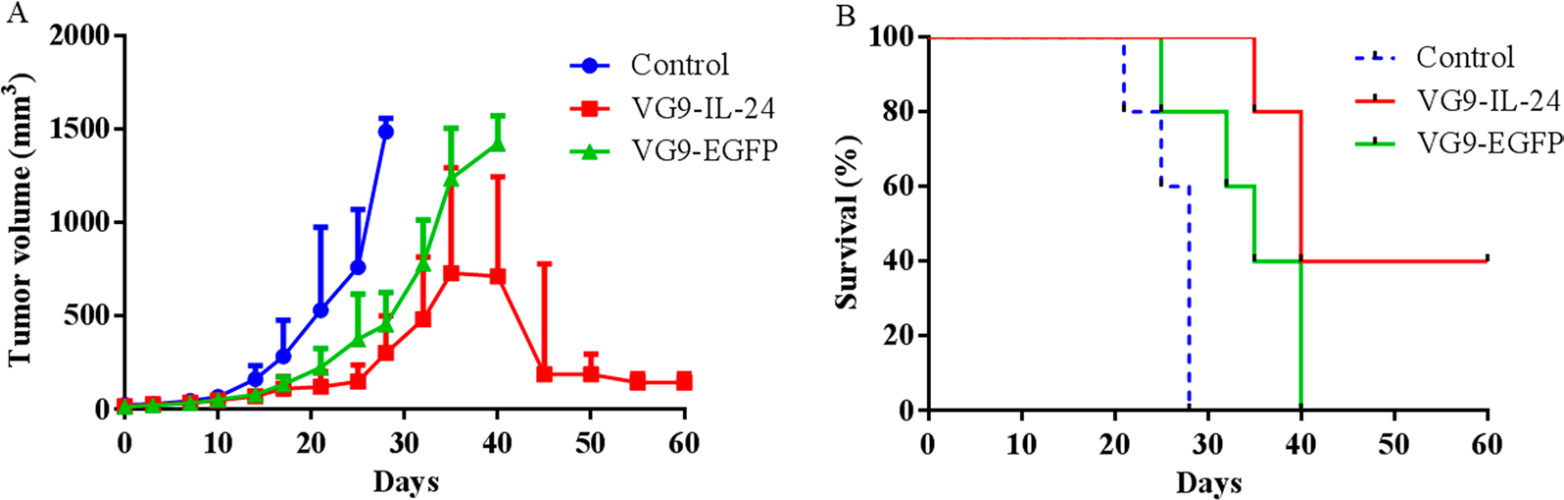
Antitumor efficacy in SMMC-7721 xenograft model in nude mice. **A** Tumor size was measured and tumor volume was monitored at various times after treatment. **B** Kaplan–Meier survival curves for tumor-bearing mice treated with PBS, VG9-IL-24, or VG9-EGFP. Log-rank test was used to analyze survival rates in the various groups. n = 6 per group

The antitumor efficacy of VG9-IL-24 was further evaluated in a subcutaneous SMMC-7721 xenograft model of nude mice. Mice bearing established tumors received a single intratumoral injection of VG9-IL-24, VG9-EGFP, or PBS (control). Results showed that by 4 weeks following the initiation of treatment, tumors in the control group had significantly increased in size [(1487 ± 71) mm^3^], whereas those in VG9-EGFP [(456 ± 172) mm^3^] or VG9-IL-24 [(304 ± 198) mm^3^] group had stabilized or regressed (Fig. 7A). A significant antitumor effect was observed in the VG9-IL-24-treated mice by 60 days post-treatment (*P* < 0.001), including two completely healing mice. Kaplan–Meier survival curves also showed that VG9-IL-24 treatment led to an improved survival rate compared to that with PBS or VG9-EGFP (both *P* < 0.05; Fig. 7B).

## Discussion

Vaccinia virus was used as the most successful live biotherapeutic agent in the campaign to eradicate smallpox. Recently, it has been emerged as a promising and appealing strategy for combating cancer owing to its appealing features. Specifically, it replicates exclusively in the cytoplasm and has a short replication cycle. Moreover, it possesses a large genome, making it capable of accepting foreign genetic insertions and achieving high levels of transgene expression by native synthetic promoters [16].

As a potential antitumor gene, IL-24 inhibits tumor growth, metastasis, invasion, and angiogenesis. The traditional delivery of IL-24 via liposomes or replication-defective adenovirus cannot target tumor cells, which limits its value for cancer gene therapy. In this study, we used vaccinia virus as a delivery vector to express IL-24 gene. Results showed that high and stable expression of IL-24 could occur with the replication of vaccinia virus, as confirmed by RT-PCR and ELISA.

Previous studies have demonstrated that IL-24 can exert its cytotoxic effects on a broad spectrum of human cancers [12, 17–21]. Our data showed that VG9-IL-24 efficiently infected and exhibited strong killing effects on various HCC cell lines. In contrast, the overexpression of IL-24 in normal live cells resulted in no significant cytotoxicity, which is similar to that reported in other studies [10–12].

As a novel cancer growth-suppressing and apoptosis-inducing gene, IL-24 plays a prominent role in inhibiting tumor growth [10, 20, 22, 23]. Hoechst staining showed that increased nuclear fragmentation, chromatin condensation, and apoptotic bodies were observed in HCC cell lines treated with VG9-IL-24. The apoptotic cell ratio was notably increased in VG9-IL-24-infected cells. Flow cytometric analyses also indicated that VG9-IL-24 induced SMMC-7721 cell cycle arrest at the G2/M phase, which is consistent with results of other studies that have demonstrated that IL-24 can induce G2/M cell-cycle arrest in various cancer cell lines [22, 24, 25].

Abundant evidences have demonstrated that the growth-inhibition properties of IL-24 are independent of the status of p53, Rb, or p21 [26, 27]. A previous study found that IL-24 expression mediated by adenovirus produced similar growth-suppressive effects on T47D (mutant p53) and MCF7 (wild-type p53) cells, as well as in MDA-MB-157 cells (null p53) [28]. In this study, comparable IL-24-induced anti-tumor activity in vitro was observed in the three HCC cell lines, of which, the cell line HepG2 expresses wild-type p53 but has a mutant Rb, whereas the cell lines SMMC-7721 and Hep3B have mutant p53 [29].

IL-24 is also an important immune mediator in addition to a tumor-suppressing agent. It exerts antitumor effects via multiple distinct pathways, including programmed cell death induction, inhibition of tumor cell invasion and metastasis, anti-angiogenic activity, immune-modulatory activity, and “bystander” antitumor activity [30, 31]. In this study, we only investigated selective apoptosis induction of VG9-IL-24. Immune competent mouse models should be established in a future study to further investigate antitumor immunity and the “bystander” antitumor effect induced by VG9-IL-24.

In terms of safety, no cytotoxicity mediated by IL-24 was previously found in a variety of normal tissue cells [9, 32]. The safety of vaccine viruses in humans has been also proven and specific antiviral agents are available [33, 34]. During our experimental period, VG9-IL-24 treatment did not cause weight loss in mice. Further, the mice were generally in good condition with no observable necrosis or ulceration of the skin, indicating that VG9-IL-24 has fewer side effects and might be clinically used.

## Conclusions

This study demonstrated that the expression of tumor-suppressing gene IL-24 mediated by vaccinia virus strain VG9 (VG9-IL-24) exhibited obvious antitumor effects on HCC both in vitro and in vivo. VG9-IL-24 could induce apoptosis in HCC cells without harming normal cells, significantly inhibited the tumor development, and improved survival. Such excellent antitumor properties of VG9-IL-24 might be due to direct cell lysis mediated by the virus, as well as selective apoptosis induction by IL-24. These findings suggest that target gene therapy mediated by vaccinia virus may provide a powerful tool for cancer treatment and VG9-IL-24 holds a significant promise as a novel strategy for HCC therapy.

## Data Availability

The datasets used and/or analysed during the current study are available from the corresponding author on reasonable request.

## Abbreviations

HCC: Hepatocellular carcinoma
GM-CSF: Granulocyte microphage colony-stimulating
TK: Thymidine kinase
IL-24: Interleukin-24
MOI: Multiplicity of infection
DMSO: Dimethyl sulfoxide
ELISA: Enzyme-linked immunosorbent assay
PI: Propidium iodide
FITC: Fluorescein isothiocyanate
